# The regulation of p53 by phosphorylation: a model for how distinct signals integrate into the p53 pathway

**DOI:** 10.18632/aging.100047

**Published:** 2009-05-07

**Authors:** Nicola J. Maclaine, Ted R. Hupp

**Affiliations:** University of Edinburgh, Institute of Genetics and Molecular Medicine, CRUK p53 Signal Transduction Laboratories, Edinburgh, EH4 2XR, Scotland, UK

**Keywords:** p53, ATM, AMPK, CK1, Ser20 phosphorylation, kinase, stress

## Abstract

The tumour suppressor p53 is a transcription factor that has evolved the ability to integrate distinct environmental signals including DNA damage, virus infection, and cytokine signaling into a common biological outcome that maintains normal cellular control. Mutations in p53 switch the cellular transcription program resulting in deregulation of the stress responses that normally maintain cell and tissue integrity. Transgenic studies in mice have indicated that changes in the specific activity of p53 can have profound effects not only on cancer development, but also on organism aging. As the specific activity of p53 is regulated at a post-translational level by sets of enzymes that mediate phosphorylation, acetylation, methylation, and ubiquitin-like modifications, it is likely that physiological modifiers of the aging function of p53 would be enzymes that catalyze such covalent modifications. We demonstrate that distinct stress-activated kinases, including ataxia telangiectasia mutated (ATM), casein kinase 1 (CK1) and AMP-activated protein kinase (AMPK), mediate phosphorylation of a key phospho-acceptor site in the p53 transactivation domain in response to diverse stresses including ionizing radiation, DNA virus infection, and elevation in the intracellular AMP/ATP ratio. As diseases linked to aging can involve activation of p53-dependent changes in cellular protective pathways, the development of specific physiological models might further shed light on the role of p53 kinases in modifying age-related diseases.

## Review

### The biochemistry and genetics of p53 function

p53 is a sequence-specific DNA-binding protein and stress-activated transcription factor that controls the expression of hundreds of genes implicated in a variety of physiological responses to genome instability, virus infection and interferon production, DNA damage, metabolic stresses such as hypoxia, and cytokine signaling. The vast numbers of gene products mediating the p53 signal coordinately promote many repair processes, some of which include elimination of damaged proteins, DNA repair, ATP generation via oxidative phosphorylation, organellar functions that maintain autophagy signaling and mitochondrial function, the cell division cycle, and programmed cell death. The implications of this stress-induced transcription re-programming by p53 is that cell and tissue integrity can be maintained, thereby contributing to organism health and viability.

**Figure 1.**
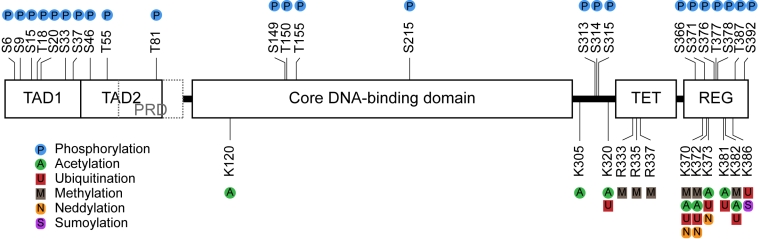
Sites of post-translational modifications on p53. Schematic representation of the 393 amino acid domain structure of human p53 showing the sites of post-translational modification including phosphorylation, acetylation, ubiquitination, methylation, neddylation, and sumoylation. Abbreviations: N-terminal transactivation domain (TAD); proline-rich domain (PRD); tetramerisation domain (TET); C-terminal regulatory domain (REG); arginine (R); lysine (K); serine (S); threonine (T).

Inactivating missense mutations in *p53 *are very common in a wide range of human cancers, indicating a critical role for p53 as a cancer suppressor in very distinct tissue microenvironments [1]. These missense mutations reside predominantly in the core DNA-binding domain or tetramerisation domain (Figure 1), and result in a p53 protein with an altered conformation and attenuated sequence-specific DNA-binding function [2]. These mutations thus suppress p53 transcription, reduce the cellular repair capacity, and stimulate tumourigenesis. As p53 is a conformationally flexible and thermodynamically unstable protein, biophysical studies have suggested there might be promise in drug developments aimed at stabilizing the mutant p53 conformation into a wild-type state, and re-engaging the p53 transcription program [3].

Transgenic technologies in mice have supported biochemical and clinical data showing a critical role for the DNA-binding function of p53 in cancer suppression. Animals null for *p53 *strikingly develop cancer at an advanced rate [4]. By contrast, deletion of many of the p53-inducible genes do not give the same tumour incidence or tumour spectrum as *p53*-null animals [5], further highlighting the role of p53 itself as a central hub in the integration of tissue repair triggers. There is one intriguing exception: animals double null for *ataxia telangiectasia mutated *(*ATM*) and the p53-inducible gene *p21 *have a similar tumour spectrum and death incidence to the *p53*-null animals [6]. This suggests that ATM and p21 form a positive genetic circuit in the p53-dependent cancer suppression mechanism.

In addition to a role for p53 in cancer control, transgenic studies have also indicated that p53 can play a role in aging-related processes that are triggered by telomere erosion or oxidative damage to proteins, lipids or DNA, which in turn affect phenotypes including neuromuscular coordination and longevity. The first such transgenic p53 animal exhibited a genetic alteration that resulted in the constitutive production of a C-terminal fragment of p53 that escaped degradation from its key negative regulator, the E3 ubiquitin ligase MDM2. These animals exhibited aging phenotypes including reduced longevity, osteoporosis, generalized organ atrophy and a diminished stress tolerance [7]. A second transgenic study showing that enhanced p53 function promotes aging utilized another truncated form of p53 with mutations in the MDM2-binding domain [8]. An additional transgenic model displaying a pro-aging phenotype had a BRCA1 mutation that constitutively activates p53 via the enhanced endogenous DNA damage signals [9]. There is also some biochemical and clinical data suggesting that p53 activation might play a role in human diseases of aging. Recent reports have shown that p53 activation can trigger the pathways that promote tau protein aggregation, which in turn is thought to reflect specific stages in Alzheimer's disease [10]. Further, the activation of p53 by β-amyloid peptides might prove *in vivo *to either suppress the accumulation of abnormal neurons by apoptotic pathways, or induce cell loss resulting in attenuated brain functions associated with aging [11].

These studies summarized above are consistent with the concept that elevated p53 activation might promote aging, which in turn seems to fit well with the models that the evolution of p53 might have come about as a trade-off between pathways that regulate longevity and maintain tissue integrity. Too much p53 might promote more efficient cancer suppression at the cost of elevated aging; whilst increasing longevity through reduced p53 function might result in elevated cancer development. However, other genetic studies that alter p53 levels have not supported these interpretations. Transgenic mice with either elevated *p53 *gene dosage [12] or hypomorphic MDM2 function [13] have no effect on the aging phenotype, although the animals have reduced cancer incidence, which would be expected if p53 function was in fact elevated.

These two distinct outcomes have been interpreted to indicate that when the *p53 *gene is under its normal physiological control, aging programs are not necessarily engaged [14]. On the other hand, artificial activation of p53 results in the abnormal production of a pro-aging phenotype, suggesting that p53 promotes aging only under abnormal or pathological circumstances [14]. This discrepancy has been resolved in part by the most recent study in which animals with enhanced *p19^ARF^* and *p53 *were generated that are under normal physiological control. These doubly transgenic mice displayed en-hanced resistance to cancer and reduced aging characteristics, including increased longevity [15], thus identifying a previously unknown anti-aging signaling trigger in vertebrates. Two key p53-inducible gene products that could play a role in this p53 anti-aging program are the antioxidants Sestrins1 and 2 whose induction by the ARF-activation signal presumably attenuates the accumulation of reactive oxygen species and associated damaged cellular constituents that would normally promote aging [16]. Thus, an understanding of the physiological factors that regulate the specific activity of p53 should shed further light on the role of p53 in aging.

**Figure 2.**
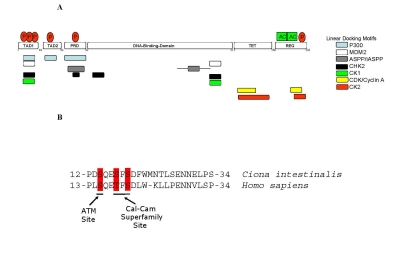
Linear Peptide Docking Sites in p53. (**A**) Linear peptide docking sites for enzymes that regulate p53 function. The N-terminus is composed of three transactivation motifs,TAD1, TAD2, and Proline-repeat domain (PRD). A key regulatory domain in the C-terminus (REG) contains the acetylation motifs and phosphorylation site and flanks the Tetramerization domain (TET). The overlapping, but distinct, linear polypeptide docking motifs for p53 regulators include the acetyltransferase p300, the E3 ubiquitin ligase MDM2, iASPP, and the protein kinases including CDK, CK2, CK1, and CHK2 are highlighted. (**B**) Conservation of key phospho-acceptor sites between urochordate and human. The panel highlights the conservation of amino acids and phospho-acceptor sites in the *BOX-I* transactivation domain of p53 (TAD1 in Figure 2A) between human and urochordate (*Ciona intestinalis*). The ATM phospho-acceptor site at Ser15 and the Calcium Calmodulin kinase/CK1 phospho-acceptors sites at Thr18 and Ser20 are highlighted as indicated.

### The biochemistry and genetics of p53 regulation

p53 protein function is regulated post-translationally by coordinated interaction with signaling proteins including protein kinases, acetyltransferases, methyl-transferses, and ubiquitin-like modifying enzymes (Figure 1). The majority of the sites of covalent modification occur at intrinsically unstructured linear peptide docking motifs that flank the DNA-binding domain of p53 which play a role in anchoring or in allosterically activating the enzymes that mediate covalent modification of p53 (Figure 2A). Such unstructured linear domains are proving to be important in signaling control [17-19]. In undamaged cells, p53 protein has a relatively short half-life and is degraded by a ubiquitin-proteasome dependent pathway through the action of E3 ubiquitin ligases including MDM2, PirH2, COP-1, and CHIP [20]. Following stress, p53 is phosphorylated at multiple residues, thereby modifying its biochemical functions required for increased activity as a transcription factor. The biochemical functions include sequence-specific DNA binding and protein-protein interactions. Acetylation of p53 is DNA-dependent, and this modification facilitates chromatin remodeling and activation of p53 target gene expression [21,22]. Of the dozens of phospho-acceptor sites reported on p53 only three (Ser15, Thr18, Ser20) are highly conserved between humans and urochordates (Figure 2B), the latter being where a bona-fide p53-MDM2 axis has appeared in evolution. Especially striking is the conservation of primary amino acid homology in the p53 transactivation domain between the invertebrate sea squirt and humans, indicating that as yet undefined evolutionary selection pressures have maintained this amino acid sequence at least since this urochordate lineage. The only other highly conserved phosphorylation site in p53 is within the C-terminus of p53 and is conserved only amongst vertebrates; the CK2 site at Ser392. As such, we have focused our research on studying two of these highly conserved phosphorylation sites in p53: the Ser20 site and the Ser392 site, as they form a paradigm to facilitate our understanding of how phosphorylation controls p53 function as a transcription factor. The many other sites of covalent modification on p53 (Figure 1) also likely play important roles in p53 function or regulation, but there are relatively smaller amounts of genetic and biochemical data describing the effects of these modifications on p53 function.

The Ser392 phospho-acceptor site is located in the C-terminal regulatory domain (REG) in a flexible and unstructured motif (Figures 1 and 2) whose phosphorylation by casein kinase 2 (CK2) stimulates the sequence-specific DNA-binding function of p53 [23]. This activation of p53 presumably occurs by changes in the conformation of the DNA binding domain that increases p53 thermostability as defined with biophysical studies using a phospho-mimetic S392D mutant p53 protein [24]. This Ser392 site is flanked by a sumoylation site [25] and a cyclin A-docking site [26]. Phosphorylation at p53 Ser392 also increases after either UV or ionizing radiation in cell lines, in mice spleenocytes *in vivo*, and in human skin basal cell populations [27-29]. These data are consistent with an activating rather than inhibitory role for phosphorylation of this site on p53 function. Critically, substitution mutation of the murine equivalent of Ser392 to Ala392 results in enhanced UV-induced skin cancer and elevated carcinogen-induced bladder cancer in transgenic mice [30,31]. These data identify a p53-activating kinase pathway whose attenuation could modify aging-related diseases in squamous tissue like skin and bladder. Whether phosphorylation of p53 at the Ser392 site plays a tumour suppressing role in other cancer types remains to be determined.

The second highly conserved phospho-acceptor site, Ser20, is located in the N-terminal transactivation domain (TAD) in an unstructured linear motif (Figures 1 and 2) whose phosphorylation stabilizes the binding of the transcriptional co-activator p300 by creating a phospho-SDLxxLL docking motif [21,22,32]. The docking of p300 to this motif is required to promote DNA-dependent acetylation of p53 at promoters, and hence transcriptional activation of p53 target genes. Mutation of Ser20 to Asp20, thereby mimicking constitutive phosphorylation of p53 Ser20, results in a p53 with enhanced transcription function in cell lines [33,34]. Further, as Ser20 site phosphorylation is elevated after DNA damage [35,36], these data suggest that phosphorylation at p53 Ser20 forms a stimulatory rather than an inhibitory signal for p53 activity. Transgenic mice with a phospho-acceptor site mutation at the Ser20 equivalent in murine p53 have been shown to develop spontaneous B-cell lymphoma [37], providing evidence of the first spontaneous cancer-prone phenotype for a p53 covalent regulatory site. Further, as B-cells from these transgenic mice exhibit attenuated ionizing radiation-induced apoptosis *in vitro* [37], these data highlight a central role for Ser20 site phosphorylation in p53-dependent apoptotic activation in this cell type.

Together, these biochemical and genetic studies show that phosphorylation can activate p53 function, although these studies do not necessarily explain what selection pressures have maintained the integrity of the Ser20 and Ser392 phospho-acceptor sites during evolution in the urochordate-chordate lineage. Nevertheless, the apparent cell- and damage-type specificity observed in post-translational modification signaling pathways highlights the need to develop tissue-specific experimental cancer models that reflect the physiological switches that can activate p53, including changes in cytokoines like transforming growth factor β (TGF-β) or interferons, metabolic stresses like hypoxia, glucose starvation or acidification, external stresses including carcinogen damage to DNA, and internal signals such as oncogene activation.

### The enzymatic pathways that regulate p53 phosphorylation at Ser20

**Figure 3.**
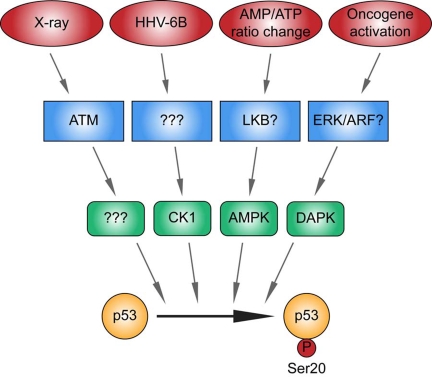
Different kinase signaling pathways link distinct stress signals to catalyze p53 phosphorylation at Ser20 in the TAD1 transactivation domain. p53 is activated by distinct stresses, some of which include as indicated, ionising radiation, viral infection, metabolic stress induced by an altered AMP/ATP ratio, and oncogene activation. The X-ray-induced Ser20 site kinase is ATM-dependent, but its identity is unknown (highlighted by "?"). CK1 is the DNA virus HHV-6B-induced p53 Ser20 kinase, but the upstream sensor is currently undefined (highlighted by "?"). The Ser20 site kinase induced by an elevated AMP/ATP ratio is AMPK, and LKB is the likely upstream sensor. DAPK-1 is the p53 Ser20 kinase induced by inappropriate oncogene activation, and ERK or ARF are the likely upstream sensors. These data support the formation of a model suggesting that the phosphorylation of p53 at Ser20 is triggered by distinct stress-responsive signaling cascades. Future analysis will be required to determine the identity of all the enzymes that mediate stress-induced phosphorylation at this site and "integrate" the p53 response and developing disease models that deregulate these signaling cascades.

Although one of the key paradigms in the p53 field is that p53 integrates diverse microenvironmental stresses into an outcome (Figure 3), the molecular mechanisms whereby these stresses activate p53 are only beginning to be defined. DNA damage activation has been the most widely studied signal input into p53. The checkpoint kinases 1 and/or 2 (CHK1/2) have been implicated as the ionizing radiation-induced p53 Ser20 site kinase(s) [38]. These enzymes have evolved an allosteric docking site in the DNA-binding domain of p53 (Figure 2A) that induces phosphorylation of p53 at Ser20 [39,40], and a second interaction site for CHK2 was identified in the proline-rich domain (PRD) of p53 [41]. Studies in transgenic mice have shown that CHK2 is required to mediate the p53-dependent response to ionizing radiation [42]. Although these data indicate CHK2 is the most likely Ser20 site kinase for p53, genetic screens have not supported this conclusion. The use of siRNA to CHK1 or CHK2 does not abrogate the damage-induced stabilization of p53 [43], and the knockout of CHK2 in cancer cell lines does not compromise Ser20 site phosphorylation [44]. Thus, the ionizing radiation-induced kinase that targets the Ser20 site of p53 is still undefined. In this study, we set out to identify the p53 Ser20 kinase(s) induced by three very different stresses that are known to activate p53: ionizing radiation, viral infection, and metabolic stress to determine whether the p53 "integration" of distinct stress signals to this phospho-acceptor site goes through the same or distinct kinase pathways.

## Results

**Figure 4.**
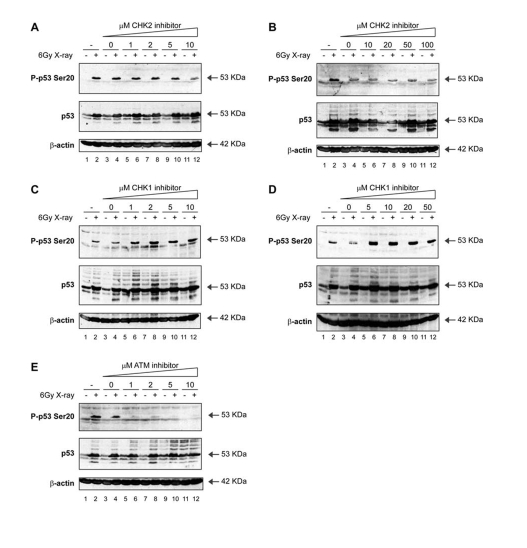
Activation of p53 by ionising radiation: effects of ATM-CHK pathway inhibitors on p53 phosphorylation. (**A, B**) A CHK2 inhibitor does not attenuate Ser20 site phosphorylation of p53 nor p53 induction mediated by treatment with X-rays. MOLT-3 cells were treated with (even-numbered lanes) or without (odd-numbered lanes) 6Gy X-ray and cultured for 4 hours after an initial 44-hour pre-treatment with: increasing concentrations [1-10μM (**A**) or 10-100μM (**B**)] of the CHK2 inhibitor (lanes 5-12), a DMSO solvent control (lanes 3-4), or a culture medium control (lanes 1-2). Cell lysates were examined by Western blotting with antibodies against the indicated proteins. (**C, D**) A CHK1 inhibitor does not attenuate Ser20 site phosphorylation of p53 nor p53 induction mediated by treatment with X-rays. MOLT-3 cells were treated with (even-numbered lanes) or without (odd-numbered lanes) 6Gy X-ray and cultured for 4 hours after an initial 44-hour pre-treatment with: increasing concentrations [1-10μM (**C**) or 5-50μM (**D**)] of the CHK1 inhibitor SB218078 (lanes 5-12), a DMSO solvent control (lanes 3-4), or a culture medium control (lanes 1-2). Cell lysates were examined by Western blotting with antibodies against the indicated proteins. (**E**) An ATM inhibitor attenuates Ser20 site phosphorylation of p53, but not p53 induction, mediated by treatment with X-rays. MOLT-3 cells were treated with (even-numbered lanes) or without (odd-numbered lanes) 6Gy X-ray and cultured for 4 hours after an initial 44-hour pre-treatment with: increasing concentrations (1-10μM) of the ATM inhibitor KU-55933 (lanes 5-12), a DMSO solvent control (lanes 3-4), or a culture medium control (lanes 1-2). Cell lysates were examined by Western blotting with antibodies against the indicated proteins.

In attempts to define whether the activation of p53 Ser20 site kinase(s) induced by different stresses is triggered by the same or different signaling pathways, we treated cells with specific kinase inhibitors in combination with distinct stresses known to activate p53. We performed all experiments using one cell culture model, namely the MOLT-3 cell line, which is a human acute lymphoblastic leukaemia T-cell line. The MOLT-3 cell line was first validated using ionizing radiation and kinase inhibitors specific for CHK2, CHK1 and ATM. As a control consistent with siRNA screens for CHK2 [43], the X-ray-induced Ser20 site phosphorylation of p53 was not attenuated by the CHK2 inhibitor (Figure 4A and B; lanes 6, 8, 10, 12 vs 5, 7, 9, 11). Further, the CHK1 inhibitor SB218078 was equally unable to prevent Ser20 site phosphorylation induced by X-rays (Figure 4C and D; lanes 6, 8, 10, 12 vs 5, 7, 9, 11). In fact, X-ray induced phosphorylation at Ser20 was elevated (Figure 4 C and D; lanes 6, 8, 10, 12 vs 4), and basal levels of p53 were stabilized by the CHK1 inhibitor in the absence X-ray treatment (Figure 4C and D; lanes 5, 7, 9, 11 vs 3). However, this stabilized form of p53 in undamaged cells was not phosphorylated at Ser20 (Figure 4C and D; lanes 5, 7, 9, 11).

These data are consistent with the recent study showing that CHK1 loss can activate p53 [45] and that CHK2 loss does not prevent Ser20 site phosphorylation [43]. Nevertheless, the treatment of cells with the specific ATM inhibitor KU-55933 resulted in a dose-dependent attenuation of X-ray-induced Ser20 site phosphorylation (Figure 4E; lanes 6, 8, 10, 12 vs 4). These data indicate that the X-ray-induced phosphorylation of p53 at Ser20 is ATM-dependent (Figure 3), but since ATM consensus sites require an SQ core motif, it is not possible for ATM to be the direct Ser20 site kinase.

**Figure 5.**
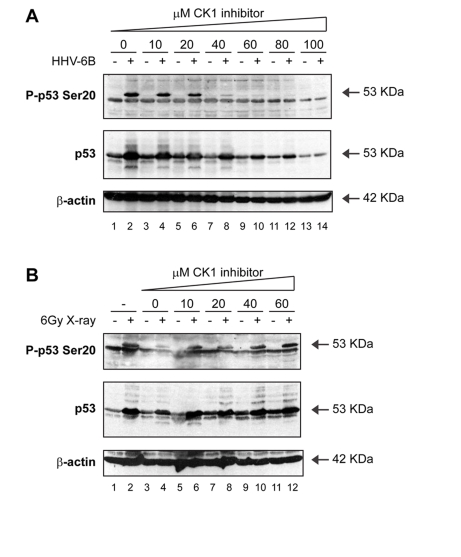
Activation of p53 by viral infection: effects of a CK1 inhibitor on p53 phosphorylation. (**A**) A CK1 inhibitor attenuates Ser20 site phosphorylation of p53 and p53 induction mediated by HHV-6B infection. MOLT-3 cells were infected with (even-numbered lanes) or without (odd-numbered lanes) HHV-6B for 48 hours in the presence of increasing concentrations (10-100μM) of the CK1 inhibitor D4476 (lanes 3-14) or a DMSO solvent control (lanes 1-2). Cell lysates were examined by Western blotting with antibodies against the indicated proteins. (**B**) A CK1 inhibitor does not attenuate Ser20 site phosphorylation of p53 nor p53 induction mediated by treatment with X-rays. MOLT-3 cells were treated with (even-numbered lanes) or without (odd-numbered lanes) 6Gy X-ray and cultured for 4 hours after an initial 44-hour pre-treatment with: increasing concentrations (10-60μM) of the CK1 inhibitor D4476 (lanes 5-12), a DMSO solvent control (lanes 3-4), or a culture medium control (lanes 1-2). Cell lysates were examined by Western blotting with antibodies against the indicated proteins.

Because the X-ray induced Ser20 kinase was still undefined, we examined whether other kinase signaling pathways, including casein kinase 1 (CK1), were involved. CK1 was identified as the human herpesvirus 6B (HHV-6B)-induced protein kinase that targets the Ser20 site on p53 [46]. Other DNA and RNA viruses are also able to activate p53 function, consistent with the intrinsic interferon-α/β responsiveness of the p53 pathway [47]. Whether these other viruses also induce p53 phosphorylation at Ser20 is not fully defined. As reported previously [46], the treatment of HHV-6B infected cells with the specific CK1 inhibitor D4476 resulted in a dose-dependent attenuation of Ser20 site phosphorylation (Figure 5A; lanes 4, 6, 8, 10, 12, 14 vs 2). However, the CK1 inhibitor had no effect on the X-ray-induced p53 Ser20 phosphorylation (Figure 5B; lanes 6, 8, 10, 12 vs 5, 7, 9, 11). Together, these data indicate that Ser20 site phosphorylation is ATM-dependent after ionizing irradiation, but CK1-dependent after virus infection (Figure 3).

**Figure 6.**
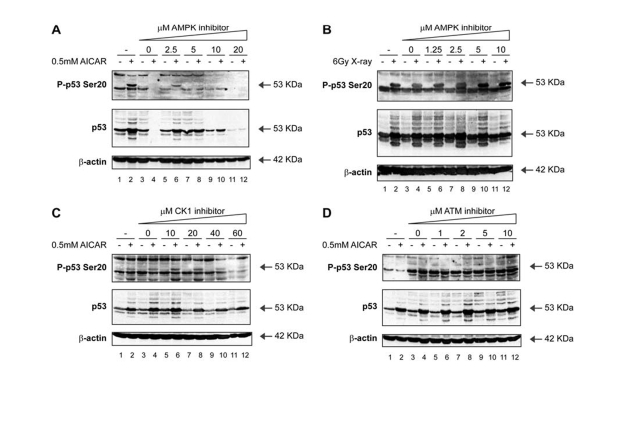
Activation of p53 by metabolic stress; effects of an AMPK inhibitor on p53 phosphorylation. (**A**) An AMPK inhibitor attenuates Ser20 site phosphorylation of p53 and p53 induction mediated by treatment with AICAR. MOLT-3 cells were treated with (even-numbered lanes) or without (odd-numbered lanes) 0.5mM AICAR for 24 hours after an initial 24-hour pre-treatment with: increasing concentrations (2.5-20μM) of the AMPK inhibitor Compound C (lanes 5-12), a DMSO solvent control (lanes 3-4), or a culture medium control (lanes 1-2). Cell lysates were examined by Western blotting with antibodies against the indicated proteins. (**B**) An AMPK inhibitor does not attenuate Ser20 site phosphorylation of p53 nor p53 induction mediated by treatment with X-rays. MOLT-3 cells were treated with (even-numbered lanes) or without (odd-numbered lanes) 6Gy X-ray and cultured for 4 hours after an initial 44-hour pre-treatment with: increasing concentrations (1.25-10μM) of the AMPK inhibitor Compound C (lanes 5-12), a DMSO solvent control (lanes 3-4), or a culture medium control (lanes 1-2). Cell lysates were examined by Western blotting with antibodies against the indicated proteins. (**C**) A CK1 inhibitor does not attenuate Ser20 site phosphorylation of p53 nor p53 induction mediated by treatment with AICAR. MOLT-3 cells were treated with (even-numbered lanes) or without (odd-numbered lanes) 0.5mM AICAR for 24 hours after an initial 24-hour pre-treatment with: increasing concentrations (10-60μM) of the CK1 inhibitor D4476 (lanes 5-12), a DMSO solvent control (lanes 3-4), or a culture medium control (lanes 1-2). Cell lysates were examined by Western blotting with antibodies against the indicated proteins. (**D**) An ATM inhibitor does not attenuate Ser20 site phosphorylation of p53 nor p53 induction mediated by treatment with AICAR. MOLT-3 cells were treated with (even-numbered lanes) or without (odd-numbered lanes) 0.5mM AICAR for 24 hours after an initial 24-hour pre-treatment with: increasing concentrations (1-10μM) of the ATM inhibitor KU-55933 (lanes 5-12), a DMSO solvent control (lanes 3-4), or a culture medium control (lanes 1-2). Cell lysates were examined by Western blotting with antibodies against the indicated proteins.

We subsequently screened cells for other signals, including hypoxia, glucose starvation, anoxia and perturbation of the AMP/ATP ratio, which could trigger p53 phosphorylation at Ser20. Of these signals, the most pronounced effect on Ser20 site phosphorylation was observed with the compound Acadesine (AICAR; Figure 6A; lane 2 vs 1), which is known to activate AMP-activated protein kinase (AMPK) by virtue of elevating the intracellular AMP levels. We had previously identified AMPK in a candidate kinase screen as an enzyme within the Calcium-Calmodulin kinase superfamily capable of targeting p53 at Ser20 *in vitro* [40]. The AICAR-mediated induction of Ser20 site phosphorylation was attenuated in a dose-dependent manner by the treatment of cells with the AMPK inhibitor Compound C (Figure 6A; lanes 6, 8, 10, 12 vs 2). On the other hand, the AMPK inhibitor was unable to prevent Ser20 site phosphorylation induced by X-rays (Figure 6B; lanes 6, 8, 10, 12 vs 5, 7, 9, 11), indicating that AMPK is not the Ser20 site enzyme induced by X-rays. Further, neither the CK1 inhibitor (Figure 6C), nor the ATM inhibitor (Figure 6D) abrogated the AICAR-induced p53 Ser20 phosphorylation (Figure 6C and D; lanes 6, 8, 10, 12 vs 5, 7, 9, 11). These data therefore confirm that p53 Ser20 phosphorylation is ATM-dependent after X-rays, CK1-dependent after virus infection, and AMPK-dependent after perturbation of AMP/ATP ratios (Figure 3).

Together, these data form a paradigm demonstrating that (i) distinct stresses, including ionising radiation, virus infection and metabolic stress in the form of altered AMP/ATP ratios, can induce p53 phosphorylation at Ser20; a site that can stabilize p300 binding [21,22,32] and whose mutation promotes the development of spontaneous B-cell lymphoma in transgenic mice [37]; and (ii) the induction of this phosphorylation depends upon distinct signals and kinase pathways, namely ATM, CK1 and AMPK (Figure 3).

## Model

### Phosphorylation in the control of p53 function

A fundamental paradigm in p53 function is that p53 "integrates" diverse stress signals towards a biological outcome. The integration mechanism is undefined but presumably involves both inhibition of p53's degradation pathway and activation of its transcription function. p53 is controlled by a variety of post-translational mechanisms (Figure 1). Of the many types of activating covalent modifications observed on p53, phosphorylation has been the most well-studied both biochemically and genetically. In this report, we have initiated a chemical biology screen to determine the mechanisms underlying the integration of stress signals to p53 activation. The fundamental question that we set out to answer is whether one common kinase pathway is able to target the Ser20 site within the transactivation domain of p53 in response to various stresses, or whether distinct kinases induced by different stresses are required to drive the same mechanism. We have focused on the Ser20 site since it is the most highly conserved phospho-acceptor site between urochordates and humans (Figure 2B) with well-documented genetic and biochemical effects. Phosphorylation at Ser20 has the most striking effect on stabilizing the p300:p53 transcription complex through interactions with multiple LxxLL peptide binding domains on p300 [21,22]. Ser20 phospho-peptides or phospho-mimetic peptides can inhibit DNA-dependent acetylation of p53, showing an important role for this modification in driving p53 acetylation [32]. Mutation of the Ser20 site equivalent in mice to Ala20 gives rise to a spontaneous tumour phenotype in transgenic animals [37], which might, in part, explain its importance, as inferred from its high conservation throughout evolution. In this study, we show that phosphorylation at the Ser20 site of p53 increases in response to distinct stresses, including ionizing radiation, virus infection or metabolic stress, and we investigate the kinase signaling pathways involved in this phosphorylation using small molecule kinase inhibitors.

### The ATM signal and aging

The phosphorylation of p53 at Ser20 after X-rays was not attributed to CHK1 or CHK2 despite original data supporting this model [38]. In addition, neither CK1 nor AMPK were the enzymes responsible for this modification. However, an ATM-dependent pathway does drive X-ray induced Ser20 site phosphorylation (Figure 3), highlighting an important clue to the identification of the X-ray-activated Ser20 site kinase. Transgenic mice with phospho-acceptor site mutations at the murine equivalent of the Ser15 ATM target site have been shown to exhibit an accelerated aging-associated phenotype, along with an enhanced spontaneous development of late-onset lymphomas [48]. This indicates that the Ser15 phospho-acceptor site is important for the tumour suppression and anti-aging activity of p53, and implies that the kinases that mediate the phosphorylation of this site, such as ATM, contribute to both the tumour suppression and anti-aging activities of p53 [48]. In a separate study, the p53 response to several forms of stress was found to decline in various tissues of aging mice [49]. In addition, the expression and activity of the kinase ATM was shown to be decreased in older mice, again highlighting the importance of this kinase for p53 function [49]. This report also suggests that decreased p53 function could, at least in part, explain the higher tumour incidence in older individuals. Finally, ATM is thought to be involved in telomere maintenance, and ATM-deficient cells undergo telomere shortening and premature senescence [50].

### The CK1 signal and aging

We had originally initiated biochemical approaches to define the Ser20 kinase induced by DNA virus infection and demonstrated that this phosphorylation is mediated by CK1 (Figure 3) [46]. Although CK1 has not generated much interest in recent years due to the fact that it is not regulated by reversible phosphorylation as are many classic stress-activated enzymes, a recent study has shown that CK1 is the major enzyme that mediates TGF-β-dependent activation of p53, however, the site of phosphorylation is at Ser6/9 in the transactivation domain [51]. As CK1 is presumably regulated by interacting proteins, it is therefore of interest to understand how stresses as distinct as virus infection or TGF-β can organize the CK1 interactome to target two different sites on p53. CK1 has also been implicated in an aging-associated disease, namely Alzheimer's disease. Indeed, the expression of CK1 has been shown to be up-regulated in the brain of Alzheimer patients [52,53], and CK1 has been implicated in the phosphorylation of the proteins tau and β-secretase that have been linked to Alzheimer's disease [54,55]. More recently, CK1 has been shown to be involved in the formation of the neurotoxic peptide amyloid-β from amyloid precursor protein [56]. Given the role of the ARF-p53 pathway in aging (reviewed in [14]) and the likelihood that cytokines like TGF-β or interferons will play tissue-specific roles in p53 modification, further examination of the role of CK1 in p53 aging models would be intriguing.

### The AMPK signal and aging

One of the key changes that occur intracellularly after stress is ATP depletion and co-incident  elevation in the ratio of AMP/ATP. The enzyme AMPK senses this change and activates a signaling cascade to reprogram the cellular response to stress. It is interesting that AMPK is the enzyme that appears to target the Ser20 site of p53 after artificially-induced changes in the AMP/ATP ratio using AICAR (Figure 3). In addition, this metabolic stress-induced Ser20 site phosphorylation is CK1- and ATM-independent, but is likely to be LKB-dependent [57]. AMPK modulates several aging-associated processes, such as mitochondrial biogenesis, obesity and decreased fatty acid oxidation, as well as insulin resistance (reviewed in [58]). In addition, AMPK activity has been shown to be decreased in aging rodent models [59,60]. AMPK dysfunction could therefore be a key factor involved in the aging-associated deficiencies in mitochondrial activity and metabolic regulation [58].

### Do kinases modify the ARF-p53 anti-aging signal?

Other cellular stresses, including aberrant oncogene activation and subsequent induction of ARF [61,62] or extracellular signal-regulated kinases (ERKs) [63] and death-associated protein kinase 1 (DAPK-1; Figure 3) [64-66] have not been evaluated as of yet due to the lack of a common cell model that has an active ARF pathway. However, given the role of ARF-p53 axis in regulating longevity (reviewed in [14]), this signal will be important to evaluate. In fact, recent studies have shown that oncogene-induced senescence does not change p53 levels but increases its specific activity [67], a phenomenon that can be accomplished by p53 phosphorylation at specific regulatory sites.

Together, these data provide a paradigm explaining how distinct stresses can activate p53 (summarized in Figure 3). In a biochemical approach to identify candidate kinases, we had previously identified many members of the calcium-calmodulin kinase superfamily, including CHK1/2, DAPK-1 and AMPK as p53 Ser20 site kinases [40]. The identification of CK1 as a major Ser20 site kinase was the first member outwith this superfamily that could target this site on p53 [46]. However, all these enzymes have a common biochemical requirement for a high affinity docking site in the core DNA-binding domain of p53 to catalyse Ser20 site phosphorylation in the transactivation domain [40,46]. Thus, cells have evolved the ability to co-opt protein kinases that respond to distinct signals to dock to the same site in the p53 DNA-binding domain and induce Ser20 site phosphorylation. The fact that many of these enzymes including ATM, CK1 and AMPK can also modify pathways in cells linked to aging phenotypes highlights a future direction for investigation aimed at understanding how these kinase signaling pathways integrate into the ARF-p53 anti-aging pathway.

## Methods

Chemicals, reagents and antibodies *. *All reagents were purchased from Sigma-Aldrich (Gillingham, UK), unless otherwise stated. The AMPK inhibitor Compound C (or Dorsomorphin), the CHK1 inhibitor SB218078, the CHK2 inhibitor, and the CK1 inhibitor D4476 were purchased from Merck Chemicals (Nottingham, UK). The ATM inhibitor KU-55933 was a gift from KuDOS Pharmaceuticals (Cambridge, UK). The DO-1 antibody to p53 was kindly provided by B. Vojtesek (Masaryk Memorial Cancer Institute, Brno, Czech Republic). P-p53 Ser20 antibody to p53 phosphorylated at Ser20 was obtained from Santa Cruz Biotechnology (supplied by Insight Biotechnology, Wembley, UK). Rabbit anti-mouse or swine anti-rabbit secondary antibodies were obtained from Dako (Ely, UK).

Cell culture and treatments. The human acute lymphoblastic leukaemia T-cell line, MOLT-3, was cultured in IMDM (Invitrogen, Paisley, UK) supplemented with 10% (v/v) foetal bovine serum (FBS; Autogen Bioclear, Calne, UK). MOLT-3 cells were infected with HHV-6B strain PL-1 as previously described [68]. Mock-infected and HHV-6B-infected MOLT-3 cells were treated with kinase inhibitors (or DMSO solvent controls) concomitantly with infection, for 48 hours. Alternatively, MOLT-3 cells were pre-treated with kinase inhibitors (or DMSO solvent controls) for 44 hours before exposure (or sham exposure) to 6Gy X-ray using a cabinet X-ray machine (Faxitron X-Ray, Illinois, USA), and further culture for 4 hours. Finally, MOLT-3 cells were pre-treated with kinase inhibitors (or DMSO solvent controls) for 24 hours before treatment with 0.5mM Acadesine (AICAR), or a DMSO solvent control, and further culture for 24 hours.

Cell lysis and Western blotting. Cells were harvested and lysed in urea lysis buffer [7M urea, 20mM HEPES (pH 7.6), 25mM NaCl, 0.05% (v/v) Triton X-100, 0.1M dithiothreitol, 5mM NaF, 2mM Na3VO4, 2.5mM Na4P2O7, and 1 x Complete Mini Protease Inhibitor Cocktail (Roche Diagnostics, Burgess Hill, UK)] by incubation on ice for 30 minutes, followed by centrifugation at 13000g for 10 minutes at 4°C. Protein lysates (40μg) were resolved by SDS-polyacrylamide gel electrophoresis (PAGE) through 10% (w/v) tris-glycine gels and transferred onto nitrocellulose membranes (Hybond ECL, GE Healthcare, Chalfont St Giles, UK). Membranes were probed with primary antibodies, followed by secondary antibodies conjugated to horse radish peroxidase (HRP). Bound antibody was detected by enhanced chemiluminescence (ECL).
