# Correction for: The regulation of p53 by phosphorylation: a model for how distinct signals Integrate into the p53 pathway

**DOI:** 10.18632/aging.206321

**Published:** 2025-09-30

**Authors:** Nicola J. Maclaine, Ted R. Hupp

**Affiliations:** 1 University of Edinburgh, Institute of Genetics and Molecular Medicine, CRUK p53 Signal Transduction Laboratories, Edinburgh EH4 2XR, Scotland, UK

**Keywords:** p53, ATM, AMPK, CK1, Ser20 phosphorylation, kinase, stress

**This article has been corrected:** The authors would like to clarify the reuse of the earlier published Western Blot images in Figure 5A. They stated: “The immunoblot data in Figure 5A was re-used from the immunoblot data in the original source article (Figure 11A) [1]. The text of the article stated that these data in Figure 5A was reported previously with reference to the source article [1]. We would now like to clarify that the immunoblot data can be re-used under the terms of the Creative Commons Attribution License (CC BY). We write this clarification to indicate that the data in Figure 5A are accurate and that this amendment does not alter the content or conclusion of this article”.

## References

[1] MacLaine NJ, Oster B, Bundgaard B, Fraser JA, Buckner C, Lazo PA, Meek DW, Höllsberg P, Hupp TR. A central role for CK1 in catalyzing phosphorylation of the p53 transactivation domain at serine 20 after HHV-6B viral infection. J Biol Chem. 2008; 283:28563–73. 10.1074/jbc.M804433200 18669630 PMC2661408

